# Measuring the Burden: Alcohol’s Evolving Impact

**DOI:** 10.35946/arcr.v35.2.02

**Published:** 2014

**Authors:** Ralph Hingson, Jürgen Rehm

**Affiliations:** **Ralph Hingson, Sc.D.,***is director of the Division of Epidemiology and Prevention Research at the National Institute on Alcohol Abuse and Alcoholism, National Institutes of Health, Rockville, Maryland.*; **Jürgen Rehm, Ph.D.,***is director of the Social and Epidemiological Research Department at the Centre for Addiction and Mental Health, chair and professor in the Dalla Lana School of Public Health, University of Toronto, Canada, and section head at the Institute for Clinical Psychology and Psychotherapy, Technische Universität Dresden, Dresden, Germany.*

**Keywords:** Alcohol use consumption, alcohol use frequency, alcohol use pattern, alcohol burden, public health impact, burden of disease, morbidity, mortality, disease, measurement, outcomes, specificity of measurement, sensitivity of measurement

## Abstract

Measuring the impact of alcohol consumption on morbidity and mortality depends on the accurate measurement of alcohol exposure, risk relationships, and outcomes. A variety of complicating factors make it difficult to measure these elements. This article reviews these factors and provides an overview of the articles that make up this special issue on current research examining alcohol’s role in the burden of disease. These topics include estimating alcohol consumption as well as alcohol-related morbidity and mortality in various demographic groups, and the burden of alcohol use disorders.

This issue of *Alcohol Research: Current Reviews* examines the public health impact of alcohol consumption beyond the role of alcohol use disorders alone (Room et al. 2005)—that is, it looks at the burden of disease. Determining impact hinges on accurate and consistent “measurements.” As demonstrated in the articles in this issue, impact typically is estimated based on three elements (Rehm et al. 2010*b*; Walter 1976):

- The measurement of exposure (i.e., the relevant dimension of alcohol use) causing the burden (Kehoe et al. 2012; Rehm et al. 2010*b*);
- The measurement of the risk relations (i.e., what level/pattern of consumption is linked to what outcome) (Rehm et al. 2010*a*); and
- The measurement of outcomes.

These different measurements are central to this issue of *Alcohol Research: Current Reviews*.

## Measurement Challenges

Numerous challenges exist when measuring the extent and predictors of alcohol-related mortality and morbidity. Those challenges also affect our ability to evaluate the effectiveness of interventions to reduce alcohol-related morbidity and mortality. Different challenges exist for acute alcohol-related mortality and chronic disease mortality and morbidity, although some of the same challenges confront measurement in both areas. The following list of challenges is illustrative but not exhaustive.

### Acute Mortality and Morbidity (Injuries and Poisonings)

Postmortem alcohol test data are not consistently available for many types of acute injury or poisoning deaths. The best available U.S. estimates indicate alcohol-attributable acute deaths outnumber chronic disease deaths 44,000 to 35,000 (Centers for Disease Control and Prevention [CDC] 2013). Traffic crashes have been the leading category of alcohol-attributable injury or death over the past 30 years. During that time period, the majority of drivers in fatal crashes (both fatally injured and, to a lesser extent, surviving drivers) have been tested for alcohol. This permits researchers to make accurate estimates of the number of drivers, passengers, and others who die in fatal crashes in which a driver was known to have been drinking.

In addition, by examining the characteristics of crashes and drivers in fatal crashes where alcohol is present, the National Highway Traffic Safety Administration (NHTSA) (Klein 1986; NHTSA 2002) has developed an imputational approach to estimate the proportion of fatal crashes involving alcohol even when the driver is not tested. This approach was verified using data from States with high percentages of drivers involved in fatal crashes that were tested for alcohol use. Researchers used the NHTSA model to predict the percentage of fatal crashes that involved alcohol and then compared those findings with the actual alcohol test results. NHTSA found they could estimate with great accuracy not only the proportion of fatal crashes involving alcohol-positive drivers but also the blood alcohol level (BAL) of the driver at the time of the crash.

Using this approach, researchers have been able to determine annual State and national estimates of alcohol involvement in fatal traffic crashes since 1982. Furthermore, having those accurate direct-test results and imputed results has permitted researchers to make epidemiologic estimates of the increased odds of fatal crashes and other crash involvement at various BALs (Voas et al. 2012). Drivers who were stopped at random in roadside surveys were compared with drivers who were fatally injured in single-vehicle crashes and who were driving in the same States on the same types of roads on the same days of the week and times of day.

Because these data are available monthly on a national, State, and community level, researchers also have been able to monitor trends in fatal crashes involving alcohol relative to fatal crashes where alcohol is not involved over time. In addition, researchers are able to use quasi-experimental and other research designs to evaluate whether State-level traffic safety legislation and community-level education and law enforcement and treatment programs are effective in reducing alcohol-related traffic deaths (Ferguson 2012; Hingson and White 2013).

**Figure 1:**
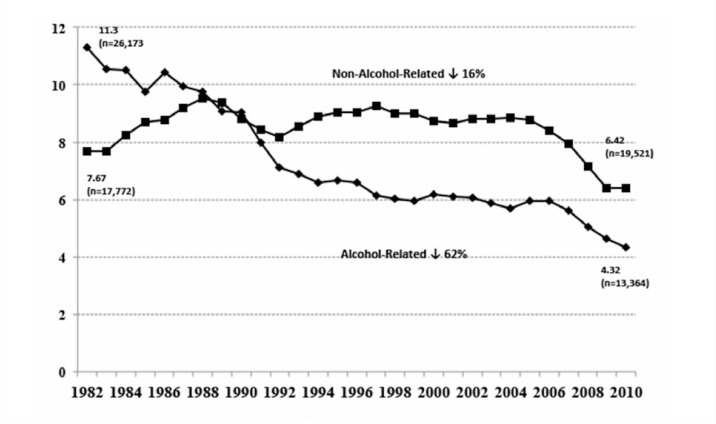
Alcohol-related versus non–alcohol-related traffic fatalities, rate per 100,000, all ages, United States, 1982–2010 SOURCES: National Highway Traffic Safety Administration, 2012; U.S. Census Bureau, 2012.

Such studies have guided policy-makers to select and implement effective programs and policies. Since the early 1980s, alcohol-related traffic death rates per 100,000 have been reduced more than 50 percent versus the decline in traffic crash death rates where alcohol is not involved (figure 1). It has been estimated that as many as 300,000 deaths have been prevented as a result of reduced incidences of drinking and driving, which is greater than those attributed to increased use of airbags, seat belts, and motorcycle and bicycle helmets combined (Cummings et al. 2006; Fell and Voas 2006).

**Figure 2:**
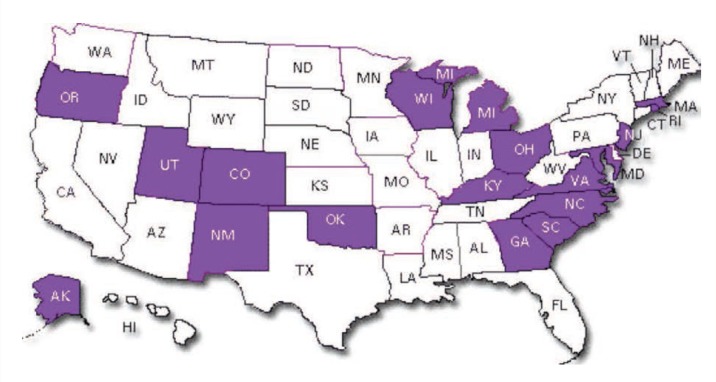
States participating in National Violent Death Registry (18 States) SOURCE: Centers for Disease Control and Prevention, National Violent Death Reporting System, 2013.

Unfortunately, unlike traffic deaths, postmortem alcohol testing is not nearly as complete for other types of unintentional or intentional poisoning and injury deaths. In 18 States (figure 2), a violent-death registry is in place where 80 percent or more of all homicides and suicides are tested. However, testing levels of alcohol for other types of injuries or deaths is not routine. As a consequence, imputations for alcohol involvement in other types of injuries or deaths have not been developed, and studies of laws and programs to reduce those types of injuries and deaths do not have the same precision as evaluations of efforts to prevent alcohol-related traffic deaths.

Second, research is emerging indicating that alcohol may interact with and pharmacologically potentiate the effects of other drugs, thereby increasing risks of motor-vehicle crashes (Asbridge et al. 2012; Li et al. 2012) and poisoning/overdose deaths (White et al. 2011). People may be involved in traffic crashes or poisoning deaths at lower BALs if other drugs are present, and this may modify BAL levels used in establishing attributable fractions for motor-vehicle and poisoning deaths. Most national surveys and many research projects inquire about alcohol and drug consumption separately not simultaneously. If alcohol and drugs pharmacologically interact, simultaneous-use questions should be considered.

Third, it is important to calculate the secondhand harm alcohol misuse poses. Just as awareness of the secondhand negative consequences of passive smoke inhalation has heightened the public health resolve to curb smoking, learning about the secondhand effects of alcohol misuse may heighten the resolve to study and implement effective interventions to reduce alcohol misuse. For example, 40 percent of people who die in traffic crashes involving drinking drivers in the United States are not driving. Half of the deaths in crashes involving drinking drivers under the age of 25 are those other than the driver. This has incited citizen activists and policymakers to pass more than 2,000 laws at the State and Federal levels to reduce alcohol-impaired driving (Hingson et al. 2003).

Fourth, many prevention activities are implemented at the community level, and community-level data are needed to stimulate the planning and evaluation of those interventions (Hingson and White 2012). Yet most surveillance data-monitoring systems measure behavior and consequences at the State and Federal levels. Strategies are needed to either facilitate more community-level data collection or to offer technical assistance to concerned communities and researchers so that they can collect their local data using standardized questions and sampling procedures for comparison with other communities, their State, and the Nation.

### Chronic Conditions

When examining either acute-disease and chronic-disease mortality and morbidity, a variety of measurement challenges may produce underreporting. First, drinking levels reported in surveys account for only 40 to 60 percent of alcohol sales (Midanik 1982; World Health Organization [WHO] 2011). Underreporting may lead to underestimates of alcohol’s contribution to chronic disease (Meier et al. 2013). Second, survey respondents often underestimate alcohol serving sizes, particularly when consumed in containers that vary from accepted standard drink sizes. Memory may become an issue after respondents have consumed so many drinks so rapidly that they incur partial memory lapses or total blackouts. Also, the duration of time that respondents are asked to recall consumption can vary in different studies. In general, shorter time periods of recall (e.g., days and weeks) produce higher consumption, estimates than requests for monthly, yearly, or lifetime consumption. On the other hand, drinking patterns may vary over time and even in the same year, prompting recommendations to use yearly recall periods. Method or mode of data collection (i.e., face-to-face, telephone, mail, or Web based) also can influence drinking reports as well as response rates and biases in survey samples. Household-based surveys may not include groups with high levels of alcohol consumption, such as students, the homeless, or people in institutions or in inpatient alcohol treatment facilities (Meier et al. 2013; Stockwell et al. 2004). Also, unrecorded alcohol, use of alcohol in food, spillage, waste, and consumption by children and tourists may not be considered in surveys (Meier et al. 2013).

Second, especially with chronic disease, in etiology studies the time proximity of drinking data collection to disease outcome must be carefully evaluated. Although some chronic diseases may take years to develop, cessation or reduction of drinking may stop the process and reduce morbidity or mortality consequences almost immediately. This can be seen in the immediate gains in mortality and life expectancy in Russia following the Gorbachev reforms that led to a reduction of drinking (Leon et al. 1997). However, these immediate gains could be found for some chronic diseases, but not for others, such as cancer. In cohort studies, drinking patterns can vary in the same individual over time, and etiology studies vary in how often someone drank over time and/or the intervals over time when drinking was measured.

Third, maintaining high response rates in surveys and longitudinal studies has become increasingly difficult over time, particularly using telephone methods, as the percentage of the population who uses mobile phones increases. If nonresponse becomes high and disproportionately involves people with characteristics and behaviors (involving but not limited to alcohol use that influence disease and injury etiology), that may cloud our understanding of alcohol’s role in the development and progression of disease. It also can limit the ability of researchers to monitor disease and death-rate trends over time.

Fourth, both in estimates of acute and chronic conditions, attributable fractions from meta-analyses of epidemiologic studies are used to estimate alcohol’s contribution to mortality and disability. Yet, these attributable fractions may change over time. For example, the percentage of factual traffic-crash deaths that involve alcohol have dropped from 60 percent to just under 40 percent in the past 30 years (NHTSA 2012). If the most current epidemiologic studies are not used in alcohol-attributable fraction estimates, the proportion of acute and chronic disease mortality and morbidity attributed to alcohol may be inaccurate.

Fifth, when chronic disease morbidity and mortality attributions are made, the range of diseases considered may vary. Current U.S. estimates may not fully consider alcohol’s role in chronic diseases such as HIV.

## Overview of Measuring the Burden: Alcohol’s Evolving Impact

This issue of ARCR examines the methodology involved in measuring the burden of alcohol use in greater detail. Drs. Flynn and Wells (2013) provide an overview on consumption indicators; environmental background indicators such as availability information; alcohol-attributable problems; indicators for alcohol-attributable health outcomes (both chronic and acute); and, last but not least, law enforcement indicators. They also make a case for triangulating different data sources in order to come to valid conclusions as well as outline methods and statistical techniques to technically integrate these data.

Dr. Cheryl Cherpitel focuses more in depth on one of these data sources for the community in her examination of hospital emergency departments (Cherpitel 2013). She describes not only the methodologies to make use of these data, such as case-control or case-crossover designs and their potential biases, but also the use of such data to derive alcohol-attributable fractions, which is a research topic in its own right (Shield et al. 2012*a*).

The next two chapters deal with two other outcomes of alcohol use. Dr. Shield and colleagues (2013*b*) summarize findings on the impact of alcohol and chronic disease (i.e., cancer, neuropsychiatric conditions, cardiovascular, and digestive diseases). Methodologically, they discuss limitations of current techniques used to derive risk relations and consequently, attributable fractions.

Drs. Rehm and Shield (2013) focus on mortality, more specifically on global estimates of alcohol-attributable mortality for the year 2010. They report the causes of death with comprise the overwhelming majority of all alcohol-attributable deaths: cancer, liver cirrhosis, and injury. Clearly, cancer reflects the mortality-related alcohol use 15 to 20 years ago, liver cirrhosis mainly current drinking but also a bit of the history, and injury with the exception suicide mainly the level of current acute consumption (Holmes et al. 2012).

No overview on alcohol use and consequences would be complete without mentioning the efforts to enumerate alcohol-attributable economic costs. The sidebar focuses on the last attempt to estimate such costs for the United States, focusing on heavy drinking (Bouchery et al. 2010).

The first part of the volume is complimented by three sidebars. Dr. Poznyak and colleagues (2013) give insight into the WHO system to collect data on alcohol consumption, alcohol-attributable harm, and alcohol policy. Drs. Wiedermann and Frick (2013) describe the use of surveys to derive disability weights to calculate the disability-adjusted life-years. Dr. Hilton (2013) introduces an important national data bank—the NIAAA Alcohol Policy Information System—for alcohol research.

In the second part of the volume, the focus is on alcohol use and its consequences over the lifespan. It starts chronologically with use by children and adolescents (Donovan 2013; Patrick 2013). Another group, in part defined by age, is college students (White 2013). All three groups again are characterized by specific methodological problems. For example, regular quantity–frequency measures do not capture alcohol use best, as consumption tends to vary. Also, especially for high-school and younger students, a lot of research is based on cross-sectional studies, which makes causal conclusions impossible. More longitudinal studies are needed to start disentangling the web of the impact of alcohol starting from earliest consumption. Such studies may append the usual self-report measures with objective measures such as repeated BAL for college students (for example, see Thombs et al. 2009).

For children, adolescents, and college students, despite the problems with establishing causality, a number of alcohol-attributable consequences have been identified. Some of them may be further in the future, as there are links between age of onset of alcohol use and alcohol dependence or other consequences in later years in the United States (Donovan 2013; Grant and Dawson 1997). The most important consequence is alcohol-attributable death. Although this outcome is relatively infrequent, a comparison to all-cause deaths during this stage of life shows that alcohol is the most important risk factor for mortality (and serious illness) (Rehm et al. 2006).

Other groups of concern highlighted in this issue include women (Wilsnack et al. 2013) and ethnic groups (Chartier et al. 2013). Although women in all countries drink less, have less heavy-drinking occasions, and experience less alcohol-attributable harm than men (WHO 2011), this gap seems to be closing in several countries including the United States (Shield et al. 2012*b*; Wilsnack et al. 2013). As for ethnicity and race, Native Americans, Hispanics, and Blacks experience higher rates of alcohol-attributable harm than Whites in the United States. This is of course in part linked to different drinking patterns (Chartier et al. 2013; Shield et al. 2013*a*); but it also may be worsened by an interaction of socioeconomic status and alcohol (Schmidt et al. 2010). Drs. Chartier and colleagues show that more detailed studies are needed, specifically on the mechanisms of alcohol’s impacts on health consequences in different ethnicities and races.

Alcohol use disorders (AUDs) are one of the most important consequences of alcohol use. Dr. Willenbring (2013) examines some of the issues related to measuring the public health impact of AUDs and treatment. Although heavy drinking is responsible for the majority of the alcohol-attributable burden of disease and mortality (for estimates, see Rehm et al. 2013), the public health impact of interventions—from screening and brief interventions to treatment of alcohol dependence—is not fully understood (for exceptions, see McQueen et al. 2011, who found that brief interventions in the hospital setting were associated with a reduction of mortality after one year of 40 percent, or Rehm et al. 2013, who found that increases of the treatment rate to 40 percent in Europe could help avoid more than 10 percent of alcohol-attributable mortality). Again, more research is needed to understand the long-term consequences of interventions.

The issue concludes with contributions on the new *Diagnostic and Statistical Manual of Mental Disorders–IV* (Grant 2013). Notes from a special NIAAA expert panel on alcohol and chronic diseases (Breslow and Mukamal 2013) also are included and outline future research opportunities for the field.
